# Macrophage ferroptosis in diabetic foot ulcers: current evidence, mechanisms, and therapeutic implications

**DOI:** 10.3389/fimmu.2026.1952662

**Published:** 2026-10-26

**Authors:** Jingyu Lang, Haiyan Zhao, Nian Zhou, Yihe Zhao, Yifu Tie, Chunyu Yang, Gang Zhao

**Affiliations:** 1 Heilongjiang University of Chinese Medicine, Harbin, China; 2 Department of Peripheral Vascular Disease, Cangzhou Hospital of Integrated Traditional Chinese and Western Medicine, Cangzhou, China; 3 Department of Peripheral Vascular Disease, The Second Affiliated Hospital of Henan University of Chinese Medicine, Zhengzhou, China; 4 Liaoning University of Chinese Medicine Xinglin College, Shenyang, China; 5 Department of General Surgery, The Third Affiliated Hospital of Heilongjiang University of Chinese Medicine, Harbin, China; 6 Department of Peripheral Vascular Disease, The First Affiliated Hospital of Heilongjiang University of Chinese Medicine, Harbin, China

**Keywords:** diabetic foot ulcer, efferocytosis, ferroptosis, inflammatory resolution, iron metabolism, lipid peroxidation, macrophages, wound healing

## Abstract

Diabetic foot ulcers are chronic wounds characterized by persistent inflammation, defective inflammatory resolution, and impaired tissue repair. Macrophages coordinate host defense, efferocytosis, inflammatory resolution, angiogenesis, and tissue reconstruction, but the diabetic wound environment can impair their adaptation, function, and survival. Ferroptosis, an iron-dependent form of regulated cell death driven by phospholipid peroxidation, has emerged as a potential mechanism linking diabetic wound stress to impaired macrophage function and survival. This review evaluates current evidence for ferroptosis in diabetic foot ulcers and distinguishes evidence from markers and pharmacological rescue obtained at the level of whole wounds from direct evidence in macrophages. We examine how altered iron handling and ferritinophagy, lipid remodeling, impaired glutathione peroxidase 4 activity and complementary antioxidant defenses, inflammatory and immunometabolic stress, and macrophage state may increase ferroptotic susceptibility. We then consider the potential consequences for macrophage functions required for repair, efferocytosis and inflammatory resolution, and support for fibroblasts, endothelial cells, and keratinocytes across wound regions and phases. Current studies support ferroptotic stress as a therapeutically modifiable process in diabetic wounds, but direct *in situ* evidence of ferroptotic stress or death in defined macrophage populations within human diabetic foot ulcers remains limited. Its biological consequences are also likely to vary according to macrophage state and wound phase. Accordingly, therapeutic strategies should avoid indiscriminate suppression or induction of ferroptosis and instead preserve macrophage functions required for antimicrobial defense, inflammatory resolution, and tissue repair while controlling excessive iron accumulation and phospholipid peroxidation. Spatially resolved biochemical analyses, longitudinal sampling, and functional perturbation in defined macrophage populations will be needed to establish causality and guide treatment according to macrophage state and wound phase.

## 1 Introduction

Chronic wounds that fail to heal impose a substantial health burden, with diabetic foot ulcers (DFUs) representing one of the most debilitating and serious complications of diabetes mellitus. After healing, approximately 40% of DFUs recur within 1 year, nearly 60% within 3 years, and 65% within 5 years (1). Most lower limb amputations associated with diabetes are preceded by foot ulceration (2, 3). Effective management requires the coordinated use of pressure offloading, debridement, infection control, restoration of perfusion when indicated, metabolic management, and appropriate wound care (4). Despite comprehensive efforts to address major mechanical, vascular, infectious, and systemic factors that impede wound repair, some DFUs remain unhealed, with persistent inflammation, impaired angiogenesis, abnormal matrix remodeling, and incomplete epithelial closure (5, 6). This continuing failure suggests that the local wound environment is characterized by cellular, metabolic, and immune disturbances that are not fully addressed by current treatment. Defining the underlying dysregulation is important not only for explaining chronicity, but also for identifying patients who may benefit from interventions directed at specific mechanisms.

Macrophages are likely to be central to this unresolved dysregulation because they coordinate both inflammatory defense and subsequent tissue repair. During normal healing, their functions change in response to wound stage and local signals, allowing them to participate in debris clearance, inflammatory resolution, angiogenesis, and tissue reconstruction (7, 8). In diabetic wounds, however, sustained metabolic stress, oxidative injury, microbial signals, and repeated tissue damage can maintain dysfunctional macrophage states (9, 10). These macrophages may continue to sustain inflammation while failing to coordinate effective resolution and repair. The persistence of macrophages within a wound therefore does not necessarily indicate that their protective and reparative functions remain intact. Processes that impair macrophage adaptation, survival, or transition between functional states may consequently contribute to both chronic inflammation and healing failure.

Ferroptosis provides a potential mechanism through which the diabetic wound environment may impair macrophage function and survival. Ferroptosis is a regulated form of cell death driven by iron-dependent peroxidation of membrane phospholipids when antioxidant defenses are insufficient (11–13). Diabetic wounds exhibit increased lipid peroxidation, disturbed iron metabolism, and altered expression of proteins involved in ferroptosis, while pharmacological inhibition of ferroptosis improves repair in experimental models (14–16). Together, the biochemical abnormalities observed in diabetic wounds and the improvements achieved through ferroptosis inhibition support a functional role for ferroptotic stress in the pathophysiology of diabetic wounds.

At the macrophage level, ferroptosis should be distinguished from other regulated cell death pathways when interpreting cellular injury in diabetic wounds. Apoptosis generally preserves plasma membrane integrity and permits clearance, whereas pyroptosis and necroptosis can amplify inflammation through membrane rupture mediated by gasdermins or receptor-interacting protein kinase 3 (RIPK3)–mixed lineage kinase domain-like protein (MLKL), respectively; autophagy is not itself equivalent to autophagy-dependent cell death (17), yet ferritinophagy can release ferritin-bound iron and increase ferroptotic susceptibility in macrophages (18). Emerging experimental evidence suggests that manipulating macrophage ferroptosis can influence diabetic wound healing, but the direction of the effect appears to depend on the macrophage population targeted and the mode of intervention (19–21). However, much of the current evidence comes from whole wound analyses, animal models, or macrophages studied *in vitro*. Direct evidence defining when, where, and in which macrophage populations ferroptotic stress or death occurs in human DFUs remains limited.

In this review, we examine macrophage ferroptotic susceptibility as a potential link between the diabetic wound microenvironment, persistent inflammation, and impaired tissue repair. We assess the evidence for ferroptosis in DFUs, discuss the mechanisms that may increase macrophage vulnerability, and consider how ferroptotic stress in specific macrophage states could affect inflammatory resolution and wound repair. We also evaluate current therapeutic concepts and the evidence needed for clinical translation.

## 2 Methods

### 2.1 Search strategy

This narrative review drew on literature identified through PubMed and Web of Science, supplemented by Google Scholar searches and examination of reference lists in relevant articles. Search terms included combinations of the following groups: [“diabetic foot ulcer” or “DFU” or “diabetic wound”], [“macrophage” or “macrophage activation” or “macrophage polarization”], and [“ferroptosis” or “iron metabolism” or “lipid peroxidation” or “antioxidant defense” or “efferocytosis”]. These groups were combined according to the topic being examined. Additional targeted searches addressed ferritinophagy, macrophage heterogeneity, inflammatory resolution, immunometabolism, wound repair, biomaterials, and local delivery. Relevant literature on single-cell and spatial analyses, lipidomics, and iron imaging was also sought to inform the discussion of experimental approaches. Searches by article title, author, or DOI were used to locate specific studies.

The literature reviewed covered publications available through August 2026, with priority given to studies published during the preceding five years. Earlier foundational studies and studies providing important mechanistic evidence were retained without a strict lower publication-date limit.

### 2.2 Study selection and evidence assessment

Articles were selected for their relevance to ferroptosis in diabetic wounds, macrophage biology, or mechanisms and interventions discussed in this review. Primary studies provided the main basis for evaluating experimental findings, while reviews and guidelines supplied broader biological and clinical context. Studies of normal wound healing and other disease models were included when they provided relevant mechanistic, functional, or methodological context, with their applicability to DFUs considered separately.

Evidence was interpreted according to the experimental model, identification of the affected cell population, measurements of ferroptosis, pharmacological or genetic interventions, and functional outcomes. Findings from human DFU tissues, experimental diabetic wounds, cultured macrophages, and other wound cell types were distinguished. Particular attention was given to whether a study directly examined macrophages or reported changes only in whole wound tissue. Marker changes and transcriptomic associations were distinguished from functional rescue evidence. Findings from non-DFU models were used as mechanistic or methodological context rather than direct evidence of macrophage ferroptosis in DFUs.

## 3 Ferroptosis in diabetic wound healing

### 3.1 Molecular basis of ferroptosis

Ferroptosis is a regulated form of cell death driven by iron-dependent peroxidation of membrane phospholipids containing polyunsaturated fatty acids (PUFAs) (11, 12). At the biochemical level, ferroptotic susceptibility is shaped by three interconnected processes: dysregulated iron metabolism, enrichment of readily oxidized lipid substrates, and insufficient detoxification of lipid peroxides (13). This framework emphasizes that ferroptosis cannot be established by a single marker but reflects the convergence of iron availability, membrane lipid composition, and antioxidant capacity.

Iron availability is a central determinant of ferroptotic susceptibility (22). Expansion of the intracellular labile iron pool (LIP) increases radical formation through Fenton reactions and promotes lipid peroxide production (22, 23). Cellular iron balance is regulated by iron uptake through transferrin receptor 1 (TfR1), iron storage in ferritin, ferritinophagy mediated by nuclear receptor coactivator 4 (NCOA4), and iron export through ferroportin (FPN) (24–26). When iron influx or the release of ferritin-bound iron exceeds the capacity for safe sequestration and export, redox-active iron accumulates and favors the progression of oxidative stress toward iron-dependent lipid peroxidation (22, 23, 26).

The lipid component of ferroptotic susceptibility is determined largely by the abundance and oxidation state of phospholipids containing PUFAs (12, 27). Acyl-CoA synthetase long-chain family member 4 (ACSL4) activates PUFAs to form PUFA-CoA, whereas lysophosphatidylcholine acyltransferase 3 (LPCAT3) incorporates these acyl chains into membrane phospholipids (27–29). These phospholipids constitute a substrate pool that is readily oxidized during ferroptosis. When radical generation increases, phospholipids containing PUFAs can be oxidized to phospholipid hydroperoxides (PLOOH), which propagate membrane lipid peroxidation and compromise membrane integrity when they are not efficiently detoxified (23, 30, 31).

Antioxidant defenses determine whether lipid peroxide accumulation remains reversible or progresses toward ferroptotic cell death (13, 31). The System Xc⁻–glutathione (GSH)–glutathione peroxidase 4 (GPX4) axis is the canonical protective pathway against ferroptosis (27, 30). System Xc⁻ imports cystine through its solute carrier family 7 member 11 (SLC7A11, also known as xCT) subunit to support GSH synthesis, while GPX4 uses GSH to reduce PLOOH to nontoxic lipid alcohols (30–32). Nuclear factor erythroid 2-related factor 2 (NRF2) further supports this defense network by regulating genes involved in antioxidant defense and ferroptosis suppression, including SLC7A11, glutamate-cysteine ligase subunits (GCLC/GCLM), and GPX4 (33). In addition to the canonical pathway mediated by GPX4, GPX4-independent mechanisms, including the ferroptosis suppressor protein 1 (FSP1)–coenzyme Q10 (CoQ10) system and pathways involving tetrahydrobiopterin, provide complementary protection against lipid peroxidation (34–36).

For the interpretation of wound studies, it is important to distinguish ferroptotic susceptibility, ferroptotic stress, and terminal ferroptotic cell death (37). This distinction is particularly important in DFUs, where markers associated with ferroptosis, such as lipid peroxidation products or altered components of the GPX4 axis, should not be interpreted alone as definitive evidence of ferroptotic death in a specific cell type. The following sections therefore distinguish general evidence of ferroptotic stress in diabetic wounds from evidence that identifies the affected cell types and more directly implicates macrophages. The principal molecular pathways governing ferroptotic susceptibility and execution are summarized in **Figure 1**.

**Figure 1:**
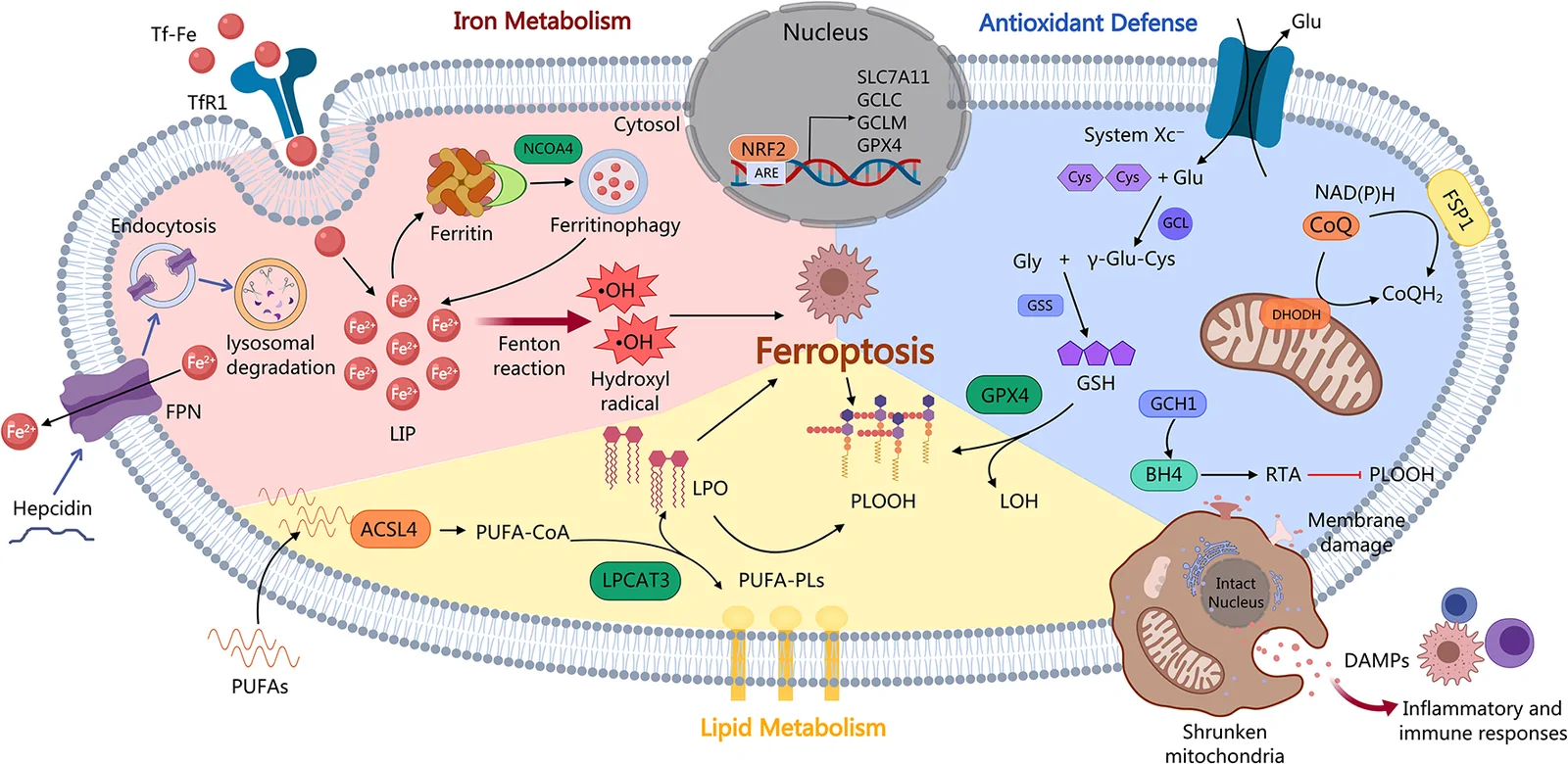
Molecular mechanisms underlying ferroptosis. Ferroptotic susceptibility is regulated by interconnected pathways involving iron metabolism, phospholipid peroxidation, and antioxidant defense. Transferrin-bound iron is internalized through transferrin receptor 1 (TfR1), whereas ferritin storage, nuclear receptor coactivator 4 (NCOA4)-mediated ferritinophagy, and ferroportin (FPN)-dependent iron export regulate the intracellular labile iron pool (LIP). Hepcidin limits iron export by promoting FPN internalization and degradation. Expansion of the LIP promotes hydroxyl radical formation through Fenton chemistry and facilitates phospholipid peroxidation. Acyl-CoA synthetase long-chain family member 4 (ACSL4) activates polyunsaturated fatty acids (PUFAs) to form PUFA-CoA, and lysophosphatidylcholine acyltransferase 3 (LPCAT3) incorporates these acyl chains into membrane phospholipids. Oxidation of PUFA-containing phospholipids generates phospholipid hydroperoxides (PLOOH). System Xc⁻ supports glutathione (GSH) synthesis by importing cystine, and glutathione peroxidase 4 (GPX4) uses GSH to reduce PLOOH to phospholipid alcohols (PLOH). Nuclear factor erythroid 2-related factor 2 (NRF2) supports antioxidant defense by regulating genes involved in cystine transport, GSH synthesis, and lipid peroxide detoxification. Complementary pathways involving ferroptosis suppressor protein 1 (FSP1), dihydroorotate dehydrogenase (DHODH), coenzyme Q, and the GTP cyclohydrolase 1 (GCH1)–tetrahydrobiopterin (BH4) system further suppress lipid radical propagation. Failure of these protective systems permits phospholipid peroxide accumulation and may culminate in ferroptotic cell death, accompanied by mitochondrial alterations, membrane damage, damage-associated molecular pattern release, and context-dependent inflammatory and immune responses. ACSL4, acyl-CoA synthetase long-chain family member 4; ARE, antioxidant response element; BH4, tetrahydrobiopterin; CoQ, coenzyme Q; CoQH_2_, reduced coenzyme Q; DAMPs, damage-associated molecular patterns; DHODH, dihydroorotate dehydrogenase; FPN, ferroportin; FSP1, ferroptosis suppressor protein 1; GCH1, GTP cyclohydrolase 1; GCLC, glutamate-cysteine ligase catalytic subunit; GCLM, glutamate-cysteine ligase modifier subunit; GPX4, glutathione peroxidase 4; GSH, glutathione; GSS, glutathione synthetase; LIP, labile iron pool; LPCAT3, lysophosphatidylcholine acyltransferase 3; NCOA4, nuclear receptor coactivator 4; NRF2, nuclear factor erythroid 2-related factor 2; PLOH, phospholipid alcohol; PLOOH, phospholipid hydroperoxide; PUFA, polyunsaturated fatty acid; ROS, reactive oxygen species; RTA, radical-trapping antioxidant; SLC7A11, solute carrier family 7 member 11; TfR1, transferrin receptor 1.

### 3.2 Evidence supporting ferroptosis in diabetic foot ulcers

Evidence supporting the involvement of ferroptosis in DFUs comes from three complementary sources: marker studies, functional intervention experiments, and transcriptomic analyses of human DFU tissues. These sources do not provide the same level of evidence. Marker studies suggest that DFUs contain an oxidative environment compatible with ferroptotic stress, whereas pharmacological rescue experiments provide stronger evidence that ferroptosis contributes to impaired wound repair. More specifically, Jiang et al. (14) reported increased 4-hydroxynonenal (4-HNE), malondialdehyde (MDA), and reactive oxygen species (ROS) in DFU wound exudate, while Li et al. (15) and Wang et al. (19) combined measurements of iron accumulation, lipid peroxidation, and ferroptosis-related proteins with ferrostatin-1 rescue in high glucose cell models and experimental diabetic wounds. Because lipid peroxidation products and changes in the expression of proteins involved in ferroptosis are not individually specific for ferroptotic cell death, findings from marker studies should be distinguished from functional rescue evidence (38).

The available clinical marker data provide supportive but not definitive evidence. A prospective clinical study reported higher levels of several indicators associated with ferroptosis in DFU samples than in traumatic wound controls, and these indicators were associated with ulcer severity and healing outcomes (14). *In vitro* and animal studies have also reported iron accumulation, increased lipid peroxidation, and altered expression of proteins involved in ferroptosis in endothelial cells exposed to high glucose, keratinocytes exposed to advanced glycation end products (AGEs), skin from diabetic mice, and experimental diabetic wound models (39, 40). Taken together, the clinical observation and experimental findings support the presence of an oxidative environment compatible with ferroptotic stress in diabetic wounds. However, they do not establish that iron-dependent phospholipid peroxidation drives cell death in a defined wound cell population. Proposed indicators in DFU tissue or wound exudate, including MDA, 4-HNE, redox status, and proteins involved in iron metabolism, therefore require further clinical validation (41).

Functional intervention studies provide stronger support for a pathogenic contribution of ferroptosis to delayed diabetic wound repair. In a diabetic wound model, Li et al. reported changes consistent with ferroptosis together with inflammation and delayed healing, while pharmacological inhibition of ferroptosis with ferrostatin-1 attenuated lipid peroxidation and improved wound repair (15). More recent studies have extended this principle by targeting antioxidant or metabolic pathways that regulate ferroptosis. For example, NRF2 activation suppressed ferroptosis and improved wound repair in diabetic mice (16). The improvement observed after ferroptosis inhibition or NRF2 activation indicates that ferroptotic stress is therapeutically modifiable in diabetic wounds, although the evidence remains primarily at the level of the whole wound (15, 16).

Human transcriptomic studies provide an additional but primarily associative layer of evidence. The single-cell transcriptomic landscape reported by Theocharidis et al. provides a clinical resource for examining dysregulated epithelial, stromal, endothelial, and immune cell states in DFU tissues (42). Subsequent single-cell studies have further characterized impaired epidermal differentiation and pathological wound microenvironments in DFU tissues (43, 44). However, enrichment of gene sets associated with ferroptosis cannot directly demonstrate lipid peroxide accumulation, the accumulation of redox-active iron, or ferroptotic cell death. These datasets are therefore best viewed as resources for hypothesis generation, with the resulting hypotheses requiring spatial, biochemical, lipidomic, and functional validation.

Taken together, current evidence supports the presence and functional relevance of ferroptotic stress in DFUs, based on evidence of oxidative lipid injury, impaired antioxidant defense, and improved healing after ferroptosis inhibition. Representative studies and their principal evidentiary limitations are summarized in **Table 1**. However, much of this evidence is derived from whole wounds or reflects pathway changes without resolving the cellular pathophysiology of ferroptotic injury. Establishing which wound cell populations are affected is therefore necessary before considering mechanisms involving macrophages.

**Table 1:** Representative studies evaluating ferroptosis in diabetic foot ulcers and experimental diabetic wounds.

Study	Experimental model	Cell type	Ferroptosis-related markers	Intervention	Key findings	Main limitations
Wang et al. (19)	Elevated glucose-exposed RAW264.7 macrophages and full-thickness wounds in diabetic rats	Macrophages *in vitro*; whole wound tissue *in vivo*	Iron, MDA, GSH, GPX4, SLC7A11, TfR1, FPN1, mitochondrial morphology, and NRF2	Ferrostatin-1; NRF2 modulation	Ferrostatin-1 reduced macrophage ferroptotic injury and inflammation and improved wound repair through an NRF2-associated mechanism.	Direct macrophage evidence was mainly *in vitro*; the improvement *in vivo* cannot be attributed exclusively to macrophage protection.
Zhong et al. (20)	Single cell transcriptomic analysis of human DFUs; macrophages exposed to high glucose and LPS; diabetic mouse wounds	Macrophages *in vitro* and in experimental wounds; human macrophage states identified transcriptomically	Lipid peroxidation, iron overload, GPX4, SLC7A11, NINJ1, and STAT3	NINJ1 manipulation; Ruan Jian Qing Mai formula	NINJ1–STAT3 signaling promoted macrophage ferroptotic injury; pathway suppression reduced inflammation, improved macrophage support for angiogenesis, and accelerated healing.	Human evidence was transcriptomic rather than biochemical proof of ferroptotic death; the therapeutic formula contains multiple active components.
Cui et al. (21)	Elevated glucose- or LPS-exposed RAW264.7 macrophages and full-thickness wounds in db/db mice	High-glucose- or LPS-stimulated RAW264.7 macrophages; F4/80^+^CD86^+^ wound macrophages classified as M1 in the study	Fe²^+^, FPN1, FTH1, NCOA4, GPX4, SLC7A11, ROS, and ferritinophagy markers	Asiaticoside; DFO and FPN1 or ferritinophagy modulation; asiaticoside-loaded GelMA	Asiaticoside promoted ferroptosis through iron retention and ferritinophagy in cultured macrophages; asiaticoside-loaded GelMA reduced wound inflammation and accelerated healing.	Cell attribution *in vivo* relied mainly on macrophage and polarization markers; changes in F4/80^+^CD86^+^ abundance did not by themselves establish state-selective ferroptosis.
Wu et al. (45)	Primary keratinocytes and HaCaT cells exposed to high glucose; diabetic rat wounds; limited human skin and wound-exudate analyses	Keratinocytes	MDA, 4-HNE, lipid oxidation, ferrous iron, ACSL4, GPX4, and mitochondrial morphology	Ferrostatin-1; ACSL4 manipulation; YTHDF2 knockdown	YTHDF2 regulated ACSL4 mRNA stability, and ACSL4-dependent ferroptosis impaired keratinocyte migration and diabetic wound healing.	Human observations were limited and did not establish ferroptotic death in intact DFU tissue; no macrophage assessment was performed.
Zhao and Liu (48)	NET-exposed fibroblasts and experimental diabetic wounds	Fibroblasts	Iron and lipid peroxidation readouts, antioxidant-pathway markers, and IRE1α/XBP1-mediated ER stress	NET and IRE1α/XBP1 pathway modulation	NETs promoted fibroblast ferroptosis through IRE1α/XBP1-mediated ER stress, reduced collagen secretion, and impaired healing.	The work used experimental models; the extent and spatial distribution of fibroblast ferroptosis in human DFUs remain unknown.
Li et al. (114)	Diabetic rat wound model with systemic pharmacological and lentiviral interventions	Not cell resolved; whole ulcer tissue	Perls’ iron staining, GPX4, SLC7A11, and inflammatory cytokines	Liproxstatin-1; circ_0005654 knockdown; IGF2BP2 overexpression	Liproxstatin-1 or circ_0005654 knockdown reduced iron deposition and inflammation, increased GPX4 and SLC7A11, and improved healing; IGF2BP2 overexpression attenuated the effect of circ_0005654 knockdown.	Animal-only study without cellular experiments or cell attribution; lipid peroxidation was not directly measured.
Jiang et al. (14)	Prospective clinical cohort: wound exudate from 59 patients with DFUs and 42 patients with traumatic wounds	Not cell resolved; wound exudate	4-HNE, MDA, and ROS	None	All three indicators were higher in DFUs and were associated with diabetes severity and healing outcomes.	Observational study at a single center; the measured products are not specific for ferroptosis and cannot identify the affected cell type.
Li et al. (15)	High glucose exposed fibroblasts and endothelial cells; STZ induced diabetic rat wounds	Fibroblasts and endothelial cells *in vitro*; whole wound tissue *in vivo*	ROS, MDA, GPX4, SLC7A11, and TfR1	Fer-1	Fer-1 reduced oxidative injury and inflammatory responses, improved cellular function, and accelerated diabetic wound healing.	Macrophages were not directly investigated. The *in vivo* findings were obtained from whole wound tissue and cannot be attributed specifically to macrophages.

Studies include direct macrophage evidence as well as evidence from other wound cells, whole wound tissue, and human DFU samples. Animal models reproduce selected features of diabetic wound healing and may not fully recapitulate the complexity of human DFUs.

4-HNE, 4-hydroxynonenal; ACSL4, acyl-CoA synthetase long-chain family member 4; CD86, cluster of differentiation 86; DFO, deferoxamine; DFU, diabetic foot ulcer; ER, endoplasmic reticulum; F4/80, murine macrophage marker; Fer-1, ferrostatin-1; FPN1, ferroportin 1; FTH1, ferritin heavy chain 1; GelMA, gelatin methacryloyl; GPX4, glutathione peroxidase 4; GSH, glutathione; IGF2BP2, insulin-like growth factor 2 mRNA-binding protein 2; IRE1α, inositol-requiring enzyme 1 alpha; LPS, lipopolysaccharide; MDA, malondialdehyde; NCOA4, nuclear receptor coactivator 4; NET, neutrophil extracellular trap; NINJ1, nerve injury-induced protein 1; NRF2, nuclear factor erythroid 2-related factor 2; ROS, reactive oxygen species; SLC7A11, solute carrier family 7 member 11; STAT3, signal transducer and activator of transcription 3; STZ, streptozotocin; TfR1, transferrin receptor 1; XBP1, X-box binding protein 1; YTHDF2, YTH N6-methyladenosine RNA binding protein 2.

### 3.3 Ferroptosis in specific cell types during diabetic wound healing

A perspective centered on individual cell types is needed because the cell populations involved in diabetic wound repair may differ in their susceptibility to ferroptosis, the functional consequences of ferroptotic injury, and the strength of the supporting evidence. In keratinocytes, stress induced by high glucose and susceptibility to ferroptosis mediated by ACSL4 have been associated with impaired proliferation and migration, suggesting a potential contribution to delayed re-epithelialization (45). In endothelial cells, exposure to advanced glycation end products or high glucose has been associated with endothelial dysfunction and ferroptotic stress, whereas inhibition of ferroptosis improves angiogenic repair in diabetic wound models (46, 47). Endoplasmic reticulum stress induced by neutrophil extracellular traps (NETs) has been reported to promote ferroptosis in fibroblasts and impair diabetic wound healing (48). Across these cell types, ferroptotic injury may compromise epithelial closure, vascular repair, and fibroblast functions required for matrix formation through distinct mechanisms.

Evidence concerning macrophages requires separate interpretation. Direct *in situ* evidence that macrophages undergo ferroptotic death in DFU tissues remains limited, but macrophages occupy a distinct regulatory position in diabetic wounds (9, 20). They integrate inflammatory and redox signals, regulate iron handling and efferocytosis, and communicate with keratinocytes, fibroblasts, endothelial cells, and neutrophils (7–9). Single-cell transcriptomic datasets from human DFUs provide a useful framework for examining dysregulated macrophage states and intercellular communication in the diabetic wound microenvironment (42–44). However, transcriptional signatures alone cannot establish iron-dependent phospholipid peroxidation or ferroptotic cell death in a defined macrophage subset. Macrophages are relevant both as a cell population that may be vulnerable to ferroptosis and as mediators of the effects of diabetic metabolic and inflammatory cues on inflammatory resolution and tissue repair.

## 4 Macrophages in diabetic wound healing

### 4.1 Functional heterogeneity of wound macrophages

Macrophages are central regulators of cutaneous wound healing because their functions change dynamically throughout the inflammatory, resolution, and repair phases (7, 8). During the early phase of wound healing, they contribute to host defense and the clearance of damaged tissue; during subsequent repair, they acquire programs that support inflammatory resolution and tissue reconstruction. In skin wounds, macrophages include both resident populations and cells derived from recruited monocytes, and their phenotypes are shaped by local cues within the wound microenvironment (7).

This functional diversity cannot be fully captured by a simple M1/M2 dichotomy. Transcriptomic and experimental studies indicate that macrophage activation comprises a spectrum of context-dependent programs rather than two mutually exclusive states (49, 50). More recent work supports a mosaic model of macrophage activation, in which human macrophages integrate multiple environmental cues and display mixed transcriptional features (51). This concept is particularly relevant to diabetic wounds, where macrophages are exposed simultaneously to hyperglycemia, advanced glycation end products, oxidative stress, microbial products, tissue damage, and altered matrix cues.

Therefore, wound macrophages are better understood as plastic cells whose states are shaped by environmental cues rather than as fixed inflammatory or reparative subsets. In normally healing wounds, this plasticity allows macrophages to coordinate the transition from inflammatory defense to clearance, resolution, and tissue reconstruction. In diabetic wounds, however, persistent metabolic and inflammatory signals can redirect this plasticity toward maladaptive activation states, biasing macrophages toward prolonged inflammatory activity while limiting factors that promote anti-inflammation and support repair (9, 10). The contrasting functional trajectories of macrophages during physiological and diabetic wound healing are illustrated in **Figure 2**.

**Figure 2:**
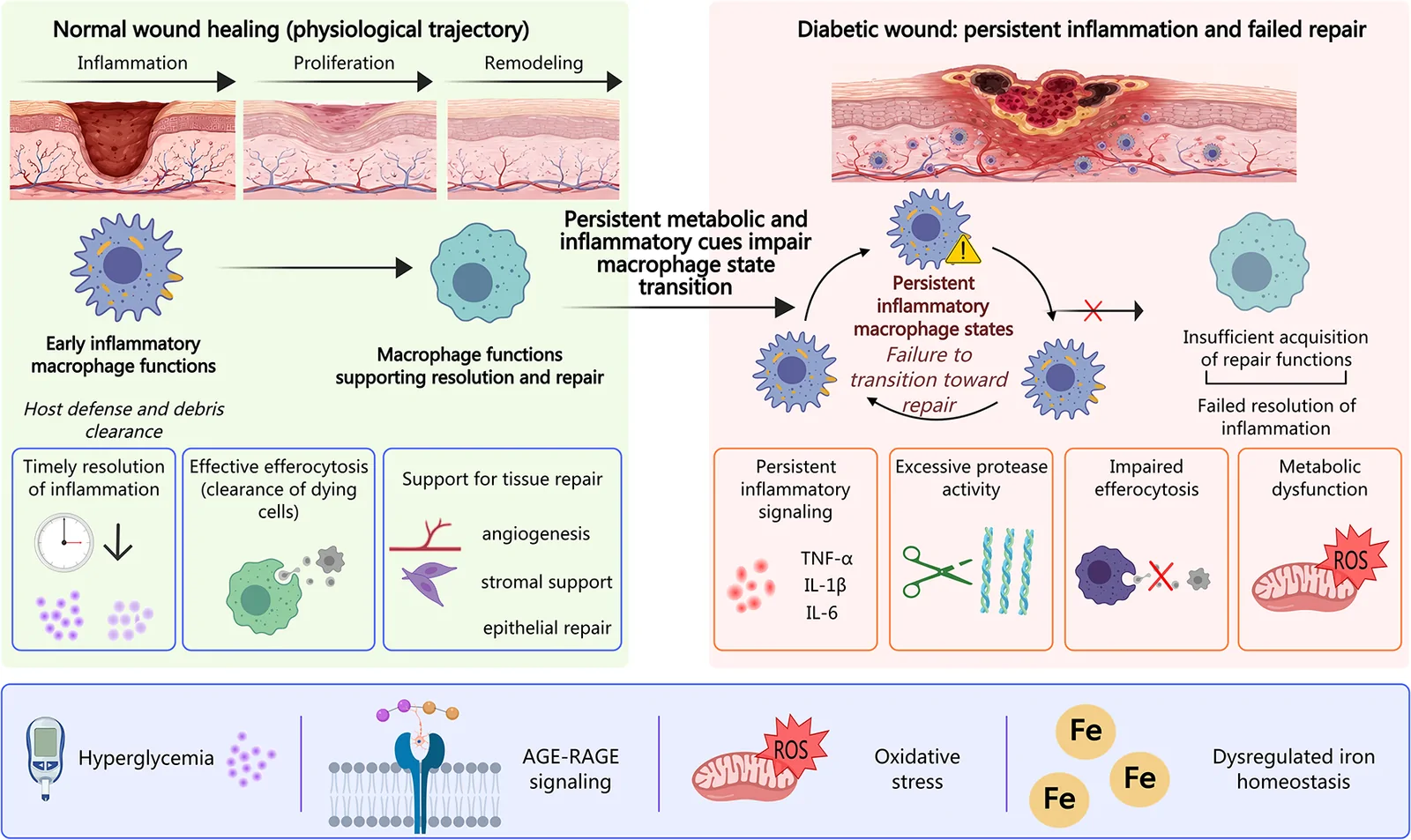
Functional trajectories of macrophages during normal and diabetic wound healing. During physiological wound healing, macrophages undergo a coordinated functional transition from early inflammatory activities to states that support inflammatory resolution and tissue repair. Early macrophage functions contribute to host defense and the clearance of damaged tissue, whereas subsequent functions promote timely resolution of inflammation, effective efferocytosis, angiogenesis, stromal support, and epithelial repair. In diabetic wounds, persistent metabolic and inflammatory cues impair macrophage state transition, resulting in persistent inflammatory macrophage states and insufficient acquisition of repair functions. Persistent inflammatory signaling, excessive protease activity, impaired efferocytosis, and metabolic dysfunction contribute to impaired inflammatory resolution and defective tissue repair. Hyperglycemia, advanced glycation end product–receptor for advanced glycation end product signaling, oxidative stress, and dysregulated iron homeostasis form an amplifying background that further constrains macrophage functional adaptation. AGE, advanced glycation end product; IL-1β, interleukin-1 beta; IL-6, interleukin-6; RAGE, receptor for advanced glycation end products; ROS, reactive oxygen species; TNF-α, tumor necrosis factor alpha.

### 4.2 Macrophage dysfunction in diabetic wounds

Diabetic wounds are characterized by dysregulated macrophage activation rather than the predominance of a single macrophage subtype. Persistent hyperglycemia, advanced glycation end products, infection, products released by biofilms, oxidative stress, and repeated tissue injury can sustain macrophage inflammatory programs that fail to resolve appropriately (9, 10). The resulting imbalance, characterized by persistent inflammatory activity and insufficient repair functions, is accompanied by sustained expression of pro-inflammatory cytokines such as IL-1β and TNF-α and chemokines such as CXCL1 and CXCL10, which can sustain leukocyte recruitment and local inflammation while limiting support for tissue repair (52). In experimental diabetic wounds, blocking interleukin-1β promotes a macrophage phenotype associated with healing and improves repair, supporting the view that sustained inflammatory signaling contributes to macrophage dysfunction and healing arrest (53).

Defective efferocytosis is another key feature of macrophage dysfunction in diabetic wounds. Efferocytosis is the clearance of apoptotic cells by macrophages, particularly apoptotic neutrophils. Beyond removing dying cells, it promotes anti-inflammatory responses and macrophage programs that support resolution and repair (54–56). In diabetic wounds, macrophage efferocytosis is impaired, uncleared apoptotic cells accumulate within the wound bed, and inflammatory resolution is delayed (57). More recent work has linked NET accumulation in diabetic wounds to impaired macrophage efferocytosis through suppression of Rac1 activation. Restoration of Rac1 activity improved efferocytosis, reduced the retention of apoptotic cells, and accelerated wound repair (58).

Several mechanisms in diabetic wounds may aggravate this clearance defect. Methylglyoxal has been reported to impair macrophage efferocytosis by promoting ROS-mediated degradation of KLF4, thereby weakening macrophage programs associated with repair (59). Mechanisms associated with infection may further contribute. For example, vesicles derived from *Staphylococcus aureus* can impair cutaneous wound healing by inhibiting efferocytosis mediated by MerTK (60).

Failure to clear apoptotic cells may therefore be both a consequence and a sustaining mechanism of chronic inflammation in diabetic wounds.

Macrophages in diabetic wounds also experience metabolic and redox disturbances that can constrain functional adaptation. Hyperglycemia and carbonyl stress alter macrophage inflammatory signaling, mitochondrial function, reactive oxygen species generation, and phagocytic capacity (9, 10, 59). Instead of completing a transition toward resolution, macrophages may remain metabolically and transcriptionally locked in states that favor pro-inflammatory cytokine production, oxidative injury, and protease activity while limiting the coordination of tissue repair. This dysfunctional state helps explain why diabetic wounds often display persistent inflammation despite the presence of abundant macrophages at the wound edge.

### 4.3 Macrophage regulation of the diabetic wound microenvironment

Macrophages shape the diabetic wound microenvironment by influencing the balance among inflammatory, proteolytic, metabolic, and reparative signals. In diabetic wounds, sustained macrophage production of pro-inflammatory cytokines, chemokines, oxidants, and mediators that regulate protease activity can maintain conditions that impede the transition from inflammation to repair (9, 10). Excessive protease activity is clinically relevant because elevated levels of matrix metalloproteinase-9 have been associated with poor healing in DFUs, and chronic wound fluid can degrade growth factors (61, 62).

Macrophage dysfunction can also disrupt coordination among epithelial, stromal, and vascular repair programs. Keratinocyte migration depends on a wound environment in which inflammatory signals decline and migratory cues are restored; fibroblast matrix deposition and remodeling require balanced inflammatory signaling and controlled extracellular matrix turnover; and endothelial sprouting depends on proangiogenic signals within a permissive tissue environment (7, 8, 63, 64). In diabetic wounds, macrophages that remain inflammatory, exhibit defective clearance, or experience metabolic stress may disrupt coordination among these repair processes, contributing to delayed re-epithelialization, poor granulation tissue formation, abnormal matrix remodeling, and insufficient microvascular repair (9, 10).

Iron handling provides an additional link between macrophage biology and wound repair. Macrophages participate in iron uptake, storage, recycling, and export. Ferroportin expressed in macrophages has also been shown to support stromal cell proliferation during wound healing (65). In chronic wounds characterized by iron accumulation, inflammatory macrophage populations can impair repair, illustrating how dysregulated macrophage iron handling may intersect with inflammation and tissue dysfunction (66). Although these observations do not establish macrophage ferroptosis in DFUs, they support the view that macrophages occupy a regulatory position at the intersection of metabolic stress, persistent inflammation, iron handling, and tissue repair. These observations provide a basis for examining how the diabetic wound microenvironment may drive macrophages toward states with increased susceptibility to ferroptosis.

## 5 Molecular determinants of macrophage ferroptosis

### 5.1 Diabetic microenvironmental cues

In diabetic wounds, macrophages are exposed to metabolic and oxidative stress, microbial products, and signals released from damaged tissue (9, 10). AGE accumulation and receptor for advanced glycation end products (RAGE) activation have long been implicated in impaired diabetic wound repair, and blockade of RAGE improves wound healing in diabetic mice. Moreover, topical inhibition of AGE–RAGE signaling ameliorates macrophage dysfunction in diabetic wounds, supporting a role for this pathway in the functional impairment of macrophages (67, 68). Under diabetic conditions, AGE–RAGE signaling can amplify inflammation through nuclear factor kappa B (NF-κB), increase reactive oxygen species generation, and enhance matrix degradation, thereby sustaining oxidative and inflammatory stress within the wound (69).

Microbial and damage-associated signals add a further layer of stress. Diabetic wounds often contain bacterial products released from biofilms and signals generated by persistent tissue injury, both of which can maintain innate immune activation. Reactive oxygen species and bacterial biofilms have both been implicated in chronic diabetic wounds, and increased Toll-like receptor 4 expression and activation contribute to prolonged inflammation in diabetic wound models (70, 71). These inputs are relevant to macrophage ferroptotic susceptibility because they increase the local oxidative burden and prolong exposure to conditions that promote lipid damage.

In the context of ferroptosis, these diabetic microenvironmental cues are best interpreted as sensitizing conditions rather than direct evidence of ferroptotic death in macrophages. Their capacity to promote ferroptosis depends on whether they coincide with more specific determinants, including the accumulation of redox-active iron, the formation of phospholipid peroxides, and impairment of the GPX4 axis. Demonstrating ferroptosis would additionally require appropriate pharmacological or genetic rescue. Thus, hyperglycemia, AGE–RAGE signaling, microbial products, and oxidative stress provide environmental pressures that may increase macrophage susceptibility to ferroptosis, whereas the following sections examine the biochemical axes that determine whether these sensitizing conditions culminate in ferroptotic stress or cell death.

### 5.2 Dysregulated iron metabolism

Macrophages actively process iron within injured tissues, making iron trafficking a key determinant of their ferroptotic susceptibility. By coordinating iron uptake, storage in ferritin, ferritin turnover, and iron export through ferroportin, macrophages regulate both intracellular iron availability and local iron redistribution (72). During cutaneous repair, iron release through ferroportin expressed by macrophages supports stromal cell proliferation, indicating that macrophage iron export has functional relevance within wound tissues (65). When iron export is insufficient or iron remobilization is excessive, redox-active iron may accumulate and increase the likelihood that oxidative stress progresses toward iron-dependent lipid peroxidation.

Inflammatory signaling may promote macrophage iron retention through mechanisms that depend on hepcidin and those that do not. Hepcidin promotes ferroportin internalization and degradation, thereby reducing cellular iron efflux (25). In inflammatory settings, IL-6–STAT3 signaling can increase systemic hepcidin expression, while hepcidin production may also be induced locally during skin inflammation or infection, including in keratinocytes and myeloid cells (73–76). In parallel, NF-κB activation has been linked to transcriptional repression of SLC40A1, the gene encoding ferroportin, in macrophages (77). These mechanisms provide plausible routes through which persistent inflammation in DFUs may promote iron retention in macrophages. However, the relative contributions of circulating hepcidin, locally produced hepcidin, and repression of ferroportin within macrophages at the wound edge remain incompletely defined.

Ferritin turnover provides another mechanism through which macrophage iron availability may increase. Ferritin normally sequesters excess intracellular iron in a less reactive form, whereas ferritinophagy mediated by nuclear receptor coactivator 4 (NCOA4) releases ferritin-bound iron into the labile iron pool (26, 78). NCOA4-mediated ferritinophagy contributes to iron mobilization in macrophages under conditions of increased iron demand (79), and variation in ferritinophagy has been associated with altered intracellular iron handling and susceptibility to ferroptosis in macrophages (18). Under sustained oxidative and inflammatory stress, dysregulated ferritin turnover may therefore increase the availability of redox-active iron and lower the threshold for lipid peroxide accumulation. Evidence from inflammatory disease models further suggests that focal iron overload can generate heterogeneous susceptibility to ferroptosis among macrophages (80), while macrophage populations characterized by iron accumulation have also been implicated in chronic wound pathology (66). However, whether altered ferritinophagy contributes to ferroptotic susceptibility in macrophage populations within human DFUs remains unresolved.

Thus, iron metabolic dysregulation in DFU macrophages is best framed as a mechanism that increases ferroptotic susceptibility rather than as proof of ferroptotic cell death. Impaired iron export through ferroportin, iron retention promoted by hepcidin, repression of SLC40A1 in macrophages, and iron remobilization mediated by NCOA4 may converge to increase the availability of redox-active iron in specific macrophage states. Determining whether macrophages with iron accumulation experience ferroptotic stress or undergo ferroptotic death in DFU tissues will require cell-resolved validation that combines measurements of iron within macrophages, detection of phospholipid peroxides, assessment of GPX4 axis integrity, and rescue by interventions that target ferroptosis.

### 5.3 Lipid peroxidation and antioxidant defense

Iron retention alone is insufficient to establish macrophage ferroptotic susceptibility. Its relevance increases when redox-active iron coincides with membrane lipids susceptible to oxidation and inadequate lipid peroxide detoxification. Inflammatory activation is accompanied by changes in macrophage lipid metabolism and phospholipid remodeling. In macrophage models, ACSL4 contributes to the incorporation of arachidonic acid into phospholipids after inflammatory stimulation, whereas LPCAT3 regulates phospholipid PUFA composition and inflammatory responses involving TLR4 (81–84). The ACSL4 and LPCAT3 data suggest that inflammatory activation may reshape macrophage membrane phospholipids in a way that increases susceptibility to peroxidation, although this has not been demonstrated directly in defined macrophage subsets within DFUs. Lipid remodeling involving ACSL4 and LPCAT3 should therefore be treated as a plausible source of peroxidizable lipid substrates rather than as a validated pathway in DFU macrophages.

The capacity to detoxify lipid peroxides is equally important. Studies of diabetic wounds have reported molecular changes consistent with ferroptotic stress, together with increased lipid peroxidation, impaired antioxidant defense, and delayed repair (15, 16). In the same studies, pharmacological inhibition of ferroptosis attenuated lipid peroxidation and improved wound healing. These findings obtained at the level of the wound support the relevance of failed lipid peroxide detoxification to impaired diabetic wound repair. Evidence more directly relevant to macrophages comes from macrophage cultures exposed to high glucose and from diabetic wound models. Wang et al. (19) showed that ferrostatin-1 reduced markers associated with ferroptotic injury and inflammation and improved wound repair *in vivo*, with NRF2 activation contributing to these effects. However, these findings should still be interpreted cautiously because most available evidence remains derived from whole wounds or macrophages studied *in vitro*, rather than spatially resolved evidence that defined macrophage subsets undergo ferroptosis in human DFU tissues.

Antioxidant defense regulated by NRF2 may modify this vulnerability. NRF2 is a broad regulator of antioxidant programs and mechanisms that suppress ferroptosis, including cystine transport, glutathione synthesis, and lipid peroxide detoxification by GPX4 (33). NRF2 activation has also been associated with improved antioxidant responses and wound repair in diabetic models (85). For macrophages, insufficient NRF2 responsiveness may reduce tolerance to sustained stress caused by lipid peroxidation, whereas NRF2 activation may help preserve lipid peroxide detoxification capacity. Nevertheless, NRF2 signaling is not specific to ferroptosis. It should not be used alone as evidence that macrophage ferroptosis has been inhibited unless accompanied by more direct readouts, such as reduced phospholipid peroxide accumulation, preservation of GPX4 axis function, or rescue by an intervention that targets ferroptosis.

Additional antioxidant systems beyond the GPX4 axis may also shape macrophage tolerance to lipid peroxide pressure. Mechanisms that function independently of GPX4, including suppression of lipid radicals by FSP1 and antioxidant protection involving tetrahydrobiopterin, can limit lipid peroxidation under certain conditions (34–36). These pathways are not yet well characterized in DFU macrophages, but they caution against reducing macrophage antioxidant defense to a mechanism centered solely on GPX4.

Overall, lipid peroxidation and antioxidant defense determine whether macrophages with iron accumulation remain stressed but viable or progress to ferroptotic injury and cell death. In DFUs, diabetic oxidative pressure, altered macrophage lipid handling, insufficient activity of the GPX4 axis, and impaired compensatory antioxidant responses may together increase macrophage ferroptotic susceptibility. This model requires further validation using lipidomic analysis of macrophages, spatial detection of phospholipid peroxidation, antioxidant pathway profiling, and functional modulation of ferroptosis in systems relevant to DFU.

### 5.4 Inflammatory and immunometabolic regulation

Persistent inflammatory signaling in DFUs can shift macrophages into a state of redox and metabolic strain. AGE–RAGE signaling, activation of pattern recognition receptors, cytokine stimulation, and signals arising from tissue damage sustain transcriptional programs driven by NF-κB and pathways that generate oxidants in diabetic wounds (86, 87). In macrophages, inflammatory activation can increase oxidant generation and reshape the cellular redox environment (88). This redox strain may increase dependence on systems that detoxify lipid peroxides, particularly the GSH–GPX4 axis and the FSP1–CoQ10 pathway supported by NAD(P)H (34, 89). Inflammatory activation alone does not define ferroptosis, but it may reduce the capacity of macrophages to restrain iron-dependent lipid peroxidation when iron handling and antioxidant defenses are already compromised.

The NOD-like receptor family pyrin domain containing 3 (NLRP3) inflammasome provides an inflammatory node relevant to DFU. Human diabetic wound tissues and macrophages exposed to high glucose show increased expression of NLRP3, caspase-1, and IL-1β, supporting a link between diabetic stress and macrophage inflammasome activation (90). NLRP3 activation is not a ferroptosis marker, but it reflects an inflammatory state associated with ROS production, cytokine amplification, and metabolic stress. In macrophages exposed to diabetic cues, inflammasome activation may therefore reduce tolerance to lipid peroxidation without replacing the need for direct evidence of ferroptosis.

Metabolites that accumulate in diabetes may further sustain macrophage states characterized by inflammasome activation. In diabetic wounds, phenylpyruvate accumulation promotes NLRP3 palmitoylation, limits lysosomal NLRP3 degradation, enhances inflammasome activation, and favors pro-inflammatory macrophage polarization (91). By sustaining inflammasome activation, phenylpyruvate may increase the oxidative burden and place additional demands on lipid peroxide detoxification, thereby reducing macrophage tolerance to ferroptotic stress.

Macrophage immunometabolism also links inflammatory activation to redox buffering and lipid peroxide control. Inflammatory macrophage activation is accompanied by remodeling of central carbon metabolism, including increased glycolytic activity and altered handling of tricarboxylic acid cycle metabolites (92). Among these metabolites, succinate can enhance IL-1β production through signaling that depends on hypoxia-inducible factor 1 alpha (HIF-1α) (93). Conversely, itaconate regulates macrophage metabolic remodeling through succinate dehydrogenase inhibition and can limit inflammatory cytokine production (94). In inflammatory macrophage models, an itaconate derivative capable of entering cells reduces lipid peroxidation and ferroptosis in macrophages through NRF2 activation and preservation of GPX4 (95). These observations link immunometabolic remodeling to antioxidant defense and tolerance of lipid peroxidation, although the contribution of these pathways in DFU macrophages remains to be defined.

In infected diabetic wounds, microbial stress may add another layer of immunometabolic pressure. Macrophage glycolysis driven by intracellular bacteria has been linked to immune dysregulation in infected diabetic wound models (96). Because the metabolic effects of infection depend on context, this mechanism is most relevant to infected or heavily colonized diabetic wounds. In these settings, microbial stress may increase glycolytic demand, disrupt redox balance, and alter lipid handling, thereby placing additional pressure on defenses against ferroptosis.

Taken together, inflammatory and immunometabolic programs help determine whether macrophages can buffer iron-dependent lipid peroxidation under diabetic wound conditions. AGE–RAGE signaling, NF-κB activity, NLRP3 inflammasome activation, diabetic metabolic intermediates, glycolytic remodeling, and redox regulation mediated by immunometabolites may converge to reduce macrophage tolerance to stress caused by lipid peroxidation. This framework also explains why macrophage vulnerability is likely heterogeneous in DFU tissues: only subsets in which inflammatory activation coincides with iron retention, lipid remodeling that increases susceptibility to oxidation, and insufficient antioxidant defense would be expected to develop ferroptotic stress. Studies with cellular resolution that integrate inflammatory state, metabolic phenotype, iron status, and phospholipid peroxidation are needed to define these susceptible macrophage populations in human DFUs.

### 5.5 Macrophage state and additional regulatory mechanisms

Macrophage ferroptotic susceptibility is unlikely to be determined by iron availability, lipid peroxide burden, or the status of the GPX4 axis alone. Differences in macrophage state can markedly influence whether lipid peroxidation becomes lethal. In myeloid models deficient in GPX4, macrophages polarized by IL-4 or activated through alternative pathways showed greater vulnerability to ferroptosis than inflammatory macrophages, indicating that macrophage activation cues and compensatory stress programs can shape ferroptotic sensitivity (97). This variation in susceptibility is particularly relevant to DFUs, where macrophages occupy heterogeneous states shaped by inflammatory, metabolic, and reparative cues rather than a uniform activation program.

Post-translational regulation provides another layer of control. FSP1 suppresses ferroptosis independently of GPX4, but post-translational modification can alter its protective capacity. SUMO-specific protease 3 (SENP3), a small ubiquitin-like modifier (SUMO)-specific protease that responds to redox changes, has been reported to sensitize macrophages to ferroptosis by interacting with FSP1 and de-SUMOylating it at K162 (98). This mechanism suggests that macrophage ferroptotic susceptibility may depend not only on the expression of antioxidant proteins but also on their post-translational state, stability, and activity.

Nerve injury-induced protein 1 (NINJ1) has recently emerged as a regulator relevant to macrophages in diabetic wound healing. A study integrating single-cell transcriptomics of human DFUs, diabetic mouse wounds, and macrophages exposed to high glucose and lipopolysaccharide (LPS) reported stronger signatures associated with ferroptosis in macrophages from diabetic wounds and identified a NINJ1–STAT3–ferroptosis pathway (20). Suppression of this pathway reduced lipid peroxidation and iron overload while preserving GPX4 and SLC7A11 expression in experimental models. Because the evidence currently comes from a limited number of studies, NINJ1 should be viewed as a promising regulatory node whose relevance to macrophage ferroptosis in human DFUs requires further validation.

Together, these mechanisms help explain why macrophage ferroptotic susceptibility is likely heterogeneous in DFUs. Differences in macrophage state, post-translational regulation of FSP1 by SENP3, and signaling involving NINJ1 may alter macrophage tolerance to iron-dependent phospholipid peroxidation. These mechanisms should not be regarded as independent proof of macrophage ferroptosis. Their value lies in identifying regulatory points at which diabetic wound stress may increase susceptibility to ferroptosis in specific macrophage populations. A state-dependent framework linking macrophage ferroptotic stress to functional consequences and therapeutic strategies is summarized in **Figure 3**.

**Figure 3:**
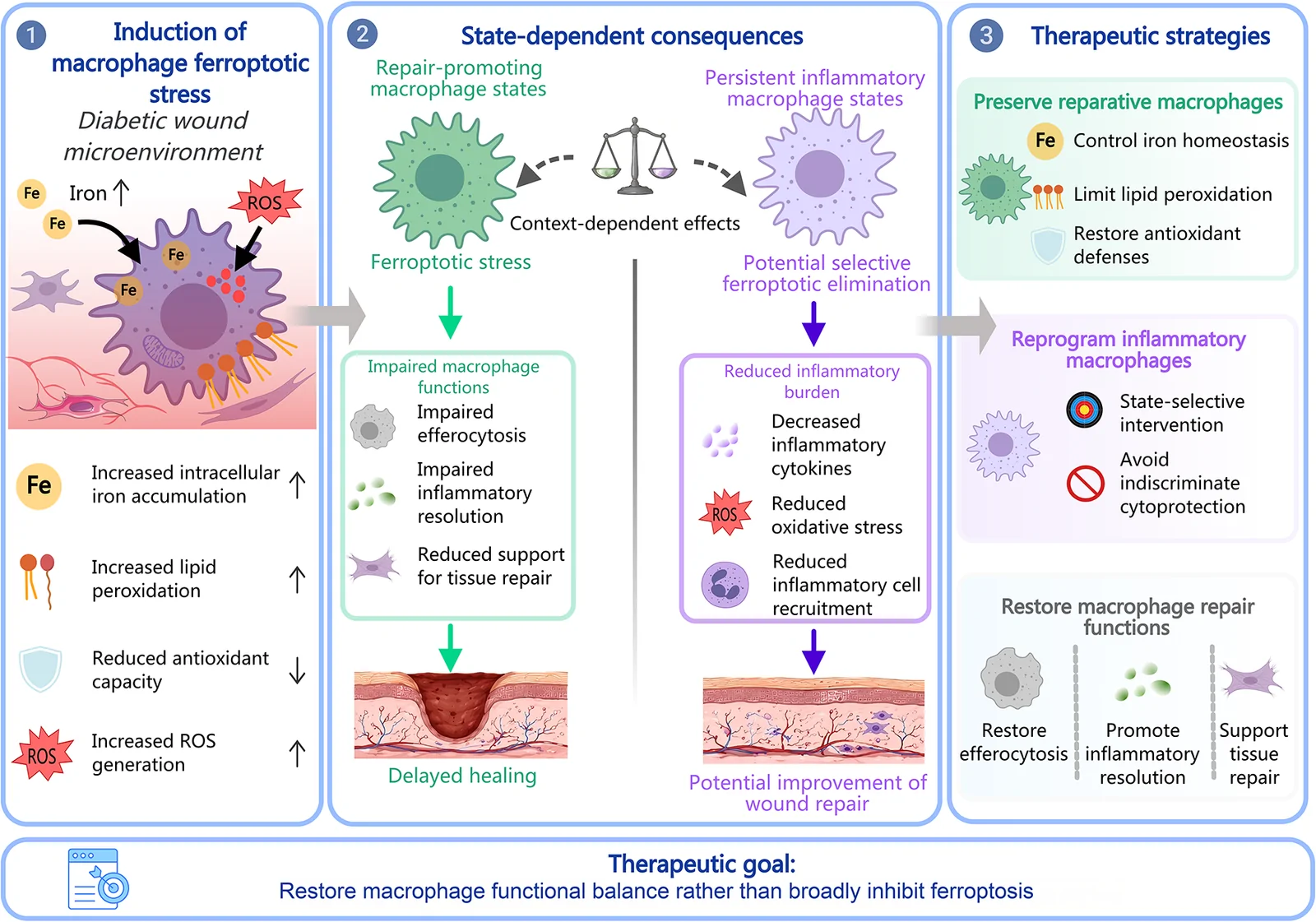
Macrophage ferroptotic stress disrupts functional balance in diabetic wounds and provides potential therapeutic opportunities. The diabetic wound microenvironment may promote ferroptotic stress in macrophages through increased intracellular iron accumulation, enhanced phospholipid peroxidation, reduced antioxidant capacity, and increased reactive oxygen species generation. The consequences of ferroptotic stress are likely to depend on macrophage functional state and wound phase. Ferroptotic loss of macrophages involved in repair could reduce the number of cells available for efferocytosis and inflammatory resolution and weaken support for tissue repair, potentially contributing to delayed healing. In some experimental settings, interventions that induce ferroptosis in cultured macrophages have also reduced inflammation and improved diabetic wound repair; however, therapeutic application would require sufficient cellular and state selectivity. Therapeutic strategies should therefore preserve reparative macrophages, reprogram persistent inflammatory macrophage states, and restore macrophage functions involved in efferocytosis, inflammatory resolution, and tissue repair rather than broadly inhibit or induce ferroptosis. ROS, reactive oxygen species.

## 6 Potential state-dependent consequences of macrophage ferroptotic injury

### 6.1 Loss of macrophage functions required for repair

The pathological consequences of macrophage ferroptotic injury in DFUs depend on which macrophage states are affected and when the injury occurs during wound repair. Macrophages are required throughout wound healing, but their functions vary across stages. Persistent predominance of macrophages with inflammatory features, together with insufficient representation of repair-associated states, is associated with prolonged inflammation and impaired healing in diabetic wounds (9). Lucas et al. (99) demonstrated that macrophage depletion during the early, middle, and late stages of skin repair produced distinct outcomes. Depletion during the early inflammatory phase significantly reduced the formation of vascularized granulation tissue, impaired epithelialization, and minimized scar formation. Depletion during the middle phase of tissue formation caused severe wound hemorrhage and prevented progression to tissue maturation and wound closure, whereas depletion during the late phase of tissue maturation did not significantly affect the outcome of the repair response. More specifically, macrophages expressing CD301b are required during the middle phase of repair, and transplantation of CD206^+^/CD301b^+^ macrophages enhances re-epithelialization, dermal cell proliferation, and fibroblast repopulation (63). Macrophage depletion and transplantation experiments show that tissue repair depends on functionally competent wound macrophage states rather than macrophage presence alone.

This focus on individual macrophage states is important because macrophage ferroptosis is not necessarily pathological in every context. The available studies that directly examine macrophage ferroptosis provide complementary but not equivalent levels of evidence. Wang et al. showed that high glucose induced iron accumulation, lipid peroxidation, and impaired antioxidant defense in cultured macrophages, and that ferrostatin-1 attenuated these changes; however, the accompanying improvement *in vivo* was assessed at the level of the whole wound (19). Zhong et al. combined human DFU single-cell data with macrophage experiments and diabetic mouse wounds to implicate a NINJ1–STAT3 pathway, providing stronger cell attribution, although the human evidence remained transcriptomic rather than direct biochemical demonstration of ferroptotic death (20). Cui et al. reported that asiaticoside induced ferroptosis in high-glucose-stimulated RAW264.7 macrophages and improved diabetic wound repair. In treated wounds, inflammation was reduced and F4/80^+^CD86^+^ macrophages, classified as M1 in that study, were less abundant; however, the reduction in this marker-defined population did not by itself establish state-selective ferroptosis *in vivo* (21). In a separate study, Chen et al. showed that SENP3-mediated de-SUMOylation of FSP1 sensitized macrophages to ferroptosis; in diabetic wounds, this pathway reduced the proportion of F4/80^+^ARG1^+^ macrophages, classified as M2 in that study, and delayed healing (98). These studies suggest that the biological consequences of macrophage ferroptosis may depend on the populations affected. Ferroptotic injury to macrophages involved in repair may weaken the cellular programs needed for inflammatory resolution and tissue formation, whereas ferroptotic loss of macrophages that sustain inflammation may reduce inflammatory activity.

Sublethal lipid peroxidation and impaired antioxidant defense may constrain the transcriptional, metabolic, and secretory programs required for repair. Because wound healing depends on macrophage states capable of coordinating epithelial, stromal, and vascular responses (63, 99), ferroptotic injury may reduce the range of functional macrophage states available for inflammatory resolution, stromal support, and tissue reconstruction. Loss of these functions provides a plausible link between macrophage ferroptotic vulnerability and delayed diabetic wound repair.

### 6.2 Impaired efferocytosis and failed inflammatory resolution

In diabetic wounds, impaired macrophage efferocytosis allows apoptotic cells to persist and contributes to delayed inflammatory resolution (57). The clearance of apoptotic cells also promotes macrophage responses that support inflammatory resolution (54, 100, 101). Loss of macrophages through ferroptosis could reduce the number of cells available for these functions. However, whether ferroptotic stress directly impairs efferocytosis in surviving macrophages in DFUs has not been established.

Studies outside DFUs have also examined how macrophages recognize and clear ferroptotic cells. Luo et al. (102) showed that oxidized phosphatidylethanolamine species exposed on ferroptotic cells act as “eat-me” signals recognized by TLR2 on macrophages, thereby promoting the phagocytic clearance of ferroptotic cells. In an *in vivo* Drosophila injury model, macrophages had difficulty engulfing ferroptosis-like necrotic cells, leading to prolonged phagocytic interactions and frequent disintegration of the dead cells; the same study also detected lipid peroxidation and 4-HNE at wound margins and 4-HNE in unengulfed cellular debris (103). In DFUs, where apoptotic cell clearance is already impaired, inefficient removal of ferroptotic cells could add to the burden of oxidized cellular remnants and contribute to persistent inflammation.

Ferroptotic cells can also release high mobility group box 1 (HMGB1), a damage-associated molecular pattern that activates inflammatory responses through AGER/RAGE signaling (104). Evidence from an inflammatory disease model further suggests that ferroptotic M2-like macrophages can release HMGB1 and stimulate TLR4–STAT3 signaling in inflammatory macrophages (105). These findings from non-DFU models support a role for HMGB1 released by ferroptotic cells in promoting macrophage inflammatory responses.

Determining whether ferroptotic stress impairs efferocytosis in DFU macrophages will require direct measurements of apoptotic cell uptake, together with assessment of macrophage viability and rescue experiments using pharmacological or genetic inhibition of ferroptosis. These experiments should distinguish reduced clearance due to macrophage loss from impaired uptake by surviving cells. Complementary spatial analyses in human DFU tissues should combine readouts of ferroptosis in macrophages with the localization of apoptotic neutrophils and ferroptotic cells, assessment of efferocytosis receptors, and characterization of macrophage programs involved in resolution.

### 6.3 Disrupted support and coordination of tissue repair

The consequences of macrophage ferroptotic injury may extend beyond macrophages by disrupting their support for fibroblasts, endothelial cells, and keratinocytes. These interactions vary across wound phases and tissue regions, so macrophage injury may not only impair individual repair pathways but also disrupt their temporal and spatial coordination. Such disruption may allow unresolved inflammation to coexist with incomplete tissue formation.

Macrophage control of stromal responses has been demonstrated in defined cell populations. Shook et al. showed that macrophages promote the proliferation of a myofibroblast subset derived from adipocyte precursors and thereby shape myofibroblast heterogeneity during skin repair (106). More recently, Honda et al. identified a functional interaction between CD206^+^ macrophages and fibroblasts expressing high levels of Gpnmb. PDGF-A released by CD206^+^ macrophages signaled through PDGFRA on fibroblasts to promote fibroblast proliferation and extracellular matrix deposition, while topical PDGF-AA restored several aspects of repair after depletion of CD206^+^ macrophages (107). Macrophage ferroptotic injury could interrupt these signals and restrict the expansion of fibroblast populations required for granulation tissue formation and matrix deposition. Whether macrophage ferroptotic injury disrupts these interactions in DFUs remains unknown.

Macrophage support for vascular repair is organized across distinct stages and vascular niches. Willenborg et al. identified a recruited CCR2+ macrophage population whose production of VEGF-A was required for effective vascularization during tissue repair (108). Live imaging by Gurevich et al. (64) showed that macrophages remained closely associated with wound vessels throughout repair but performed distinct functions at different stages. Early in repair, they promoted vessel sprouting, whereas during resolution they contributed to the regression and clearance of newly formed vessels. Macrophage ablation during early repair impaired neoangiogenesis, whereas ablation during resolution disrupted vessel regression. Ferroptotic injury in macrophages occupying these vascular niches could therefore reduce early support for endothelial sprouting and disturb the later remodeling of new vessels. In a diabetic wound model, inhibition of the NINJ1–STAT3–ferroptosis pathway reduced inflammation and improved the ability of macrophages to promote angiogenesis, whereas NINJ1 overexpression reversed these effects (20). Whether the same relationship occurs in human DFU tissues remains unknown.

Macrophage depletion studies also indicate a role in epithelial repair. Depletion of CD301b^+^ macrophages during the middle phase of skin repair delayed re-epithelialization and reduced dermal cell proliferation (63). Depletion of CD206^+^ macrophages produced a similar delay in wound closure and re-epithelialization, together with reduced fibroblast expansion and thinner granulation tissue (107). A recent roadmap of human skin wounds further showed that macrophages and fibroblasts support keratinocyte migration during different phases of repair (109). These findings suggest that ferroptotic injury to macrophages may delay epithelial closure by reducing support at stages when macrophage signals are required. However, this relationship remains inferential because the depletion studies did not examine ferroptosis and the signals through which these macrophage populations regulate keratinocyte migration remain incompletely defined.

Regional variation within wounds may further shape these effects. Integrated single cell and spatial analyses of skin wounds have identified fibroblast states that occupy distinct regions and change over time, together with corresponding changes in their communication with immune cells (110). Human DFU studies at single cell resolution have likewise revealed marked macrophage heterogeneity and fibroblast populations associated with healing (42). However, these datasets do not identify which macrophage populations undergo ferroptotic injury or whether they are spatially linked to impaired matrix formation, vascular growth, or epithelial repair. Defining these relationships will require spatially resolved analyses that combine readouts of ferroptosis in macrophages, including iron accumulation and phospholipid peroxidation, with measures of fibroblast expansion, endothelial sprouting, and keratinocyte migration in the same tissue regions (37). Combined with functional perturbation, such analyses will help determine whether macrophage ferroptosis merely accompanies the nonhealing state or contributes to the loss of coordinated tissue repair in DFUs.

## 7 Therapeutic strategies targeting macrophage ferroptotic vulnerability

Therapeutic strategies targeting macrophage ferroptosis vulnerability should address both the biochemical conditions that promote ferroptosis and the macrophage functions needed for wound repair. The aim is not to suppress ferroptosis indiscriminately or to preserve every macrophage population. Treatment should instead protect macrophages that contribute to inflammatory resolution and tissue repair by limiting excessive iron accumulation and lipid peroxidation and by restoring antioxidant defenses and the capacity to clear dying cells. When local delivery is used, cellular exposure should also be carefully controlled. Whether ferroptosis can be modulated selectively to protect macrophages supporting repair or eliminate those sustaining inflammation in diabetic wounds remains unresolved. Most current studies evaluate treatment effects in the wound as a whole. Improvements in wound closure or reductions in oxidative stress therefore do not necessarily indicate direct protection of macrophages from ferroptosis. The following sections distinguish interventions examined directly in macrophages from approaches supported by studies of whole wound tissue, other wound cell types, or general ferroptosis mechanisms. Approaches without direct macrophage evidence are discussed for their potential relevance to future treatment development, rather than as established therapies targeting macrophage ferroptosis in DFUs.

### 7.1 Regulation of iron homeostasis

Macrophages regulate iron uptake, storage, recycling, and export, making iron balance an important therapeutic consideration in DFUs. The aim should be to limit excess labile iron rather than deplete iron indiscriminately. Iron is required for immune defense, cellular metabolism, and tissue formation, and iron released from macrophages through ferroportin supports stromal cell proliferation and vascular development during wound repair (65). Excessive iron retention may increase susceptibility to lipid peroxidation, whereas excessive iron depletion could interfere with the trophic functions of macrophages and restrict tissue repair. Because macrophage iron handling is linked to systemic iron homeostasis, systemically administered chelators may alter physiological iron availability beyond the wound (22). Therapeutic regulation of iron must therefore preserve local iron trafficking and systemic iron homeostasis while limiting the accumulation of redox-active iron that promotes oxidative injury.

Deferoxamine is the most extensively investigated iron chelator in diabetic wound models. In diabetic rats, local deferoxamine treatment increased HIF-1α accumulation, improved neovascularization, and accelerated wound healing (111). An optimized transdermal formulation also increased vascularity, dermal thickness, collagen deposition, and wound strength in diabetic models (112). These studies support beneficial effects on diabetic wound repair, but the observed improvements have mainly been attributed to activation of HIF-1α–VEGF signaling and enhanced vascular responses. They did not determine whether deferoxamine reduced iron accumulation or suppressed ferroptosis in wound macrophages, or whether either effect contributed to improved diabetic wound repair. At present, deferoxamine is best regarded as a wound-level intervention rather than an established therapy targeting macrophage ferroptosis.

Interventions may also act directly on iron handling within macrophages. Maintaining iron export through ferroportin may help limit intracellular iron retention and preserve macrophage functions during wound repair (65). Limiting excessive mobilization of ferritin-bound iron represents another possible approach, although this strategy has not been tested directly in DFU macrophages (26, 78). Conversely, asiaticoside reduced FPN1 expression and promoted FTH1 degradation through ferritinophagy, resulting in intracellular Fe²^+^ accumulation and ferroptosis in cultured inflammatory macrophages. In the same study, treatment also improved diabetic wound healing (21). These contrasting approaches suggest that the therapeutic effect of iron regulation may depend on the macrophage population being targeted. Reducing iron accumulation may protect macrophages involved in resolution and tissue repair, whereas selectively promoting iron retention may be beneficial when the intervention is restricted to macrophages that sustain chronic inflammation.

Treatments directed at iron metabolism will require careful control of dose, timing, route of administration, and cellular exposure. A practical strategy may combine moderate reduction of excess labile iron with preservation of iron export through ferroportin and of macrophage functions required for tissue repair.

### 7.2 Inhibition of lipid peroxidation

Lipid peroxidation is a central execution process in ferroptotic membrane injury. Excess iron, oxidative stress, and impaired antioxidant defenses converge on the oxidation of phospholipids containing polyunsaturated fatty acids. Intercepting lipid radical propagation may therefore limit ferroptotic injury even when the upstream abnormalities are not fully corrected (12, 27). In macrophages involved in inflammatory resolution and tissue repair, reducing lipid peroxide accumulation may help preserve cell viability and function. However, the therapeutic effect will still depend on which macrophage populations are exposed and when treatment is applied.

Ferrostatin-1 and liproxstatin-1 are radical-trapping antioxidants that inhibit ferroptosis by interrupting lipid autoxidation and limiting the propagation of membrane lipid peroxidation (113). Wang et al. (19) found that ferrostatin-1 reduced iron accumulation and lipid peroxidation, restored antioxidant defenses, and attenuated inflammatory responses in RAW264.7 cells exposed to high glucose. In the same study, ferrostatin-1 reduced markers of ferroptotic stress and inflammation in diabetic rat wound tissue, promoted cell proliferation and neovascularization, and accelerated wound closure; these changes were accompanied by increased NRF2 expression. Li et al. (114) reported that liproxstatin-1 reduced iron deposition and inflammatory cytokine levels, increased GPX4 and SLC7A11 expression, and improved healing in a diabetic rat wound model. Because lipid peroxidation was not measured directly and the affected cell population was not identified, these findings are consistent with inhibition of ferroptosis at the level of whole wound tissue but do not demonstrate direct protection of macrophages. Despite these findings, ferrostatin-1 and liproxstatin-1 remain experimental inhibitors whose pharmacological specificity, potential off-target effects, and toxicity profiles in diabetic wounds require further evaluation before clinical translation (115).

Based on established mechanisms of ferroptosis, another potential therapeutic approach is to reduce the formation of phospholipids that are particularly susceptible to oxidation. ACSL4 activates polyunsaturated fatty acids, and LPCAT3 incorporates the resulting acyl chains into membrane phospholipids, thereby expanding the pool of phospholipids susceptible to peroxidation (28, 29). Targeting either step may reduce lipid peroxide accumulation. Recent diabetic wound studies have identified ferroptosis mediated by ACSL4 in keratinocytes and shown that manipulation of this pathway can alter wound healing, but corresponding evidence in DFU macrophages has not been reported (45). Broad inhibition of ACSL4 or LPCAT3 could also interfere with membrane remodeling, inflammatory lipid mediator production, and normal immune functions. Therapeutic intervention should therefore reduce pathological lipid peroxidation without disrupting the lipid metabolism required for macrophage activity and tissue repair.

### 7.3 Restoration of antioxidant defenses

Restoring endogenous antioxidant defenses differs from directly trapping lipid radicals because it aims to restore the sustained capacity of macrophages to remove phospholipid hydroperoxides. The SLC7A11–GSH–GPX4 system remains the most established basis for this approach. Maintaining cysteine and GSH availability or preserving GPX4 activity may increase resistance to ferroptotic injury, but direct therapeutic evidence in DFU macrophages remains limited (30–32). Increases in SLC7A11 or GPX4 expression in whole wound tissue provide supportive evidence but do not establish that lipid peroxidation has been reduced in macrophages or that their functions have recovered. Future therapeutic studies need to relate changes in these proteins to lipid oxidation, macrophage survival, and functional outcomes such as clearance of dying cells and inflammatory resolution.

Local activation of NRF2 provides evidence at the level of the wound that a broader antioxidant program can be restored in diabetic wounds. In db/db mice, Rabbani et al. (116) found that topical RTA 408 increased NRF2 activity and the expression of downstream antioxidant genes, reduced oxidative stress in the wound, and accelerated wound closure. Treated wounds also showed greater vascularization of granulation tissue and improved re-epithelialization. These findings support local restoration of redox balance, although the study did not examine ferroptosis or determine whether macrophages were directly protected. The broad activity of NRF2 may be therapeutically useful because it affects GSH synthesis, peroxide detoxification, and iron handling, but it also requires careful control. HO-1, for example, contributes to antioxidant defense, whereas excessive heme degradation can increase intracellular iron and promote ferroptosis under some experimental conditions (117). The effects of NRF2 activation should therefore be evaluated by measuring lipid peroxidation, iron availability, and macrophage function rather than NRF2 or HO-1 expression alone.

More direct evidence for targeting antioxidant defense in macrophages comes from the SENP3–FSP1 pathway. FSP1 maintains coenzyme Q10 in its reduced form at cellular membranes and suppresses lipid peroxidation independently of GPX4 (34). Chen et al. showed that SENP3 removed SUMO from FSP1 at K162 and increased macrophage susceptibility to ferroptosis. In diabetic wounds, SENP3 promoted ferroptosis in macrophages classified as M2 in that study and reduced their proportion, whereas loss of SENP3 improved healing (98). These findings identify preservation of FSP1 activity or inhibition of its de-SUMOylation by SENP3 as possible strategies for protecting macrophages involved in repair. However, no selective intervention targeting this pathway has yet been validated for DFUs. Other defenses, including the GCH1–tetrahydrobiopterin pathway, may also limit lipid oxidation independently of GPX4 (36), but their therapeutic relevance to DFU macrophages remains unknown. Current evidence therefore supports further evaluation of local NRF2 activation at the level of the wound and identifies the SENP3–FSP1 pathway as a more direct macrophage target. Therapeutic enhancement of GPX4, FSP1, or the GCH1–tetrahydrobiopterin pathway still requires further validation.

### 7.4 Restoration of macrophage functions

Preventing ferroptotic death may preserve macrophage numbers, but it does not necessarily restore functions already impaired by the diabetic wound environment. Therapeutic strategies should therefore also restore macrophage metabolic adaptability, efferocytosis, functions involved in inflammatory resolution, and communication with cells involved in tissue repair. Treatment should restore macrophage functions according to the stage of healing rather than impose a uniform reparative state, because macrophages also make essential contributions during the early inflammatory phase (99).

PPAR-γ is a nuclear receptor activated by ligands that regulates metabolic and inflammatory programs in macrophages. Mirza et al. found that PPAR-γ signaling normally increases as wound macrophages acquire functions involved in inflammatory resolution and tissue repair, but this response was impaired in diabetic mouse and human wounds. Myeloid deletion of PPAR-γ prolonged wound inflammation and delayed healing. Treatment with the PPAR-γ agonists 15d-PGJ2 and rosiglitazone promoted macrophage responses associated with healing, and topical administration improved wound repair in diabetic mice (118). Although ferroptosis was not examined, these findings support the view that restoring metabolic and inflammatory regulation mediated by PPAR-γ in macrophages can improve diabetic wound healing.

Restoring efferocytosis provides another route to restoring macrophage function. Topical sulforaphane accelerated healing in diabetic mice, increased macrophage clearance of apoptotic cells, and enhanced MerTK expression through NRF2 and HO-1 signaling (119). Similarly, 4-octyl itaconate improved diabetic wound repair by enhancing efferocytosis and macrophage responses that promote inflammatory resolution through the MCT1–lactate–GPR132 pathway (120). The reported effects also included changes in endothelial cells and neutrophils, indicating that the intervention influenced multiple cell populations within the wound. By restoring apoptotic cell clearance and inflammatory resolution, these interventions may complement direct ferroptosis inhibition and preserve the contribution of macrophages to tissue repair.

### 7.5 Local delivery using biomaterials

Local delivery is likely to be important for translating ferroptosis-modulating strategies into treatments for DFUs. Biomaterials can serve two complementary functions in this setting. As delivery platforms, they can retain, protect, and control the release of ferroptosis inhibitors or other cargo directed at iron homeostasis, lipid peroxidation, and antioxidant defense (121, 122). Some bioactive material systems may also exert antioxidative or other wound-modulating effects rather than serving solely as passive carriers (122). The local environment of a DFU can dilute or degrade topically applied agents, while impaired perfusion may limit the amount reaching the wound after systemic administration (123–125). Hydrogels and related dressings can retain therapeutic agents within the wound, protect unstable cargo, and control the duration of exposure. These properties are particularly relevant to ferroptosis-modulating interventions, for which local delivery may increase wound exposure while reducing systemic effects on physiological iron metabolism and antioxidant functions.

#### 7.5.1 Hydrogel biomaterials

Hydrogel design can be matched to the mechanism and cellular response being targeted. A silk nanofiber hydrogel containing deferoxamine provided sustained local delivery and improved angiogenesis and tissue repair, providing an example of local iron chelator delivery for diabetic wound treatment (126). A hydrogel responsive to matrix metalloproteinase-9 (MMP-9) released cinnamaldehyde in response to MMP-9 activity within the wound and reduced ferroptotic injury in endothelial cells, providing a model for linking drug release to local pathological signals (127). Gelatin methacryloyl (GelMA) has also been used to sustain asiaticoside delivery, reduce wound inflammation, and improve diabetic wound healing; the same study demonstrated ferroptosis induction in cultured macrophages (21). These studies show that hydrogel biomaterials can support different therapeutic aims, including protection of cells required for repair and delivery of agents that induce macrophage ferroptosis. Material design should therefore account for biocompatibility, the intended cellular target, therapeutic cargo, and stage of wound healing.

#### 7.5.2 Nanoparticle and extracellular vesicle-based delivery platforms

Nanoparticles and extracellular vesicles provide additional means of delivering agents that regulate redox balance or carry biological cargo within diabetic wounds. Zhao et al. coassembled bilirubin and morin into nanoparticles without an additional carrier and incorporated them into a collagen/polyvinyl alcohol hydrogel. The nanoparticles scavenged excess ROS and suppressed inflammatory responses *in vitro*. In diabetic mice, the composite dressing increased IL-10, reduced TNF-α, shifted macrophage markers toward a profile associated with repair, and promoted collagen maturation (128). This formulation links antioxidant nanoparticle delivery with macrophage regulation and provides a basis for testing whether local redox control can reduce ferroptotic stress in wound macrophages. Extracellular vesicles offer a biologically derived delivery platform. Yang et al. (129) provided a more direct link between this form of delivery and ferroptosis by showing that exosomes from mesenchymal stem cells (MSCs) pretreated with coenzyme Q10 transferred miR-548ai and miR-660 to keratinocytes, reduced ACSL4-mediated ferroptosis, and accelerated wound healing in diabetic mice. Although the study concerned keratinocytes rather than macrophages, it illustrates how miRNAs carried by extracellular vesicles can alter ferroptotic susceptibility in a defined wound cell population. A thermosensitive chitosan hydrogel prolonged the local release of extracellular vesicles derived from MSCs and improved wound healing in diabetic rats (130). Another hydrogel with self-healing and antibacterial properties delivered exosomes derived from bone marrow MSCs, enhanced angiogenic responses, and modulated macrophage activity in diabetic wounds (131). Collectively, these studies show that nanoparticle- and extracellular vesicle-based delivery systems can serve two related roles in diabetic wounds: delivering defined cargo to modulate ferroptosis in specific wound cell populations and regulating broader redox, inflammatory, vascular, and matrix responses.

Clinical translation of biomaterial systems that deliver ferroptosis inhibitors, exert antioxidant effects, or modulate ferroptosis through other mechanisms will require evidence beyond improved wound closure. Local retention, release kinetics, degradation, safety, and, where relevant, antimicrobial performance must be defined for each delivery system, together with the reproducibility of nanoparticle and extracellular vesicle preparations. Local delivery does not itself confer selectivity for macrophages or particular macrophage states, and the same cargo may also reach other wound cell populations. It will therefore be necessary to determine which cells are exposed to the therapeutic cargo and confirm that the intended macrophage populations show the expected changes in iron handling, phospholipid peroxidation, or function. Biomaterials may provide a practical route for local treatment, but their value will ultimately depend on reliable delivery to the relevant cells and preservation or redirection of macrophage functions according to the needs of the wound.

## 8 Current challenges and future perspectives

### 8.1 Current knowledge gaps

Direct evidence that macrophages undergo ferroptotic stress or ferroptotic death *in situ* in DFUs remains limited. Most studies have examined whole wound tissue, cultured cells, or mixed cell populations, while single-cell transcriptomic datasets identify altered macrophage states without directly demonstrating ferroptosis (42). Commonly used markers of lipid peroxidation, lipid metabolism, and antioxidant defense may indicate oxidative injury or increased susceptibility, but no single marker is sufficient to establish ferroptosis. Future studies should first identify macrophages and then, within the same tissue regions, combine measurements of labile iron, oxidized membrane phospholipids, impaired antioxidant defenses, membrane injury, and cell viability with rescue by appropriate pharmacological or genetic interventions (132). Such evidence will be needed to distinguish macrophage ferroptosis from overlapping oxidative and cellular changes caused by infection, ischemia, inflammation, and tissue necrosis.

Future work should also distinguish ferroptotic susceptibility, ferroptotic stress, functional impairment caused by sublethal lipid damage, and terminal ferroptotic death. Macrophage state and wound phase may influence both susceptibility to ferroptosis and the consequences of ferroptotic injury: loss of macrophages that sustain inflammation could reduce inflammatory activity, whereas injury to populations required for efferocytosis, resolution, and tissue repair could delay healing (133). The key questions are therefore which macrophage populations are affected, where they are located, when ferroptotic stress arises during wound progression, and when it begins to alter macrophage function. Addressing these questions will require the spatial, longitudinal, lipidomic, and functional approaches discussed in the following section.

### 8.2 Emerging technologies and experimental priorities

Single-cell RNA sequencing can identify macrophage states with altered transcriptional programs related to iron handling, lipid metabolism, and antioxidant defense. Spatial transcriptomics can then determine whether macrophage states with these transcriptional features are concentrated at the wound edge, wound bed, perivascular regions, or inflammatory niches. Human DFU studies have shown that cell populations and signals associated with healing vary across wound regions, supporting spatial analysis rather than assuming that a single biopsy represents the entire ulcer (42). Longitudinal sampling is equally important. A recent single cell multiomics map of human skin wounds showed that macrophage and stromal interactions change across inflammatory, proliferative, and remodeling phases (109). Similar analyses in DFUs could reveal whether macrophage states associated with increased ferroptotic susceptibility appear transiently or persist in nonhealing wounds.

Redox lipidomics can detect and quantify oxidized phosphatidylethanolamine species implicated in ferroptotic execution (134). Fluorescent probes can detect changes in the labile Fe(II) pool during macrophage activation and ferroptosis, while newer genetically encoded reporters enable single-cell visualization of bioactive iron and oxygen *in vivo*; adapting these approaches to diabetic wound models with macrophage labeling could map iron availability across macrophage populations and wound regions (135, 136). Metabolomic analysis could complement these measurements by assessing glutathione metabolism, cysteine availability, and other changes in redox metabolism (137). Proteomic analysis could determine whether transcriptional changes are reflected at the protein level and identify changes in proteins involved in antioxidant defense, phospholipid metabolism, ferritin turnover, and iron export (138). When combined with measurements of membrane injury, cell viability, and functional rescue, integration of these data could help distinguish a transcriptional stress program from biochemical lipid damage and ferroptotic cell death.

Profiling must be paired with functional perturbation. Macrophage-specific manipulation of GPX4, ACSL4, SLC7A11, FSP1, NCOA4, or regulators of iron handling could test whether experimentally increasing or reducing ferroptotic susceptibility alters inflammation and wound healing. Lineage tracing could determine whether affected macrophages die, change state, or are replaced. Human DFU biopsies and wound explants could provide spatial and biochemical validation of macrophage ferroptosis signatures identified in experimental models, while coculture systems could test how macrophage ferroptotic injury affects keratinocytes, fibroblasts, and endothelial cells. Rescue experiments should confirm that observed effects reflect ferroptosis rather than nonspecific antioxidant actions. Combining spatial multiomics with macrophage-specific perturbation will be important for moving from association toward mechanism.

### 8.3 Clinical translation

Clinical translation will first require biomarkers that reflect macrophage ferroptosis more specifically than generic oxidative stress. A useful panel may combine lipid peroxidation products with indicators of iron handling, antioxidant defense, macrophage abundance, and failed inflammatory resolution. Wound exudate may permit repeated sampling, whereas tissue biopsy can provide more direct cellular and spatial information. Candidate panels should be validated against tissue findings and tested prospectively for their associations with healing, recurrence, infection, and amputation outcomes.

Patient selection and treatment timing will also be critical. DFUs vary in duration, infection burden, perfusion, neuropathy, metabolic control, and the balance between active inflammation and failed repair. Protecting macrophages from ferroptosis may be beneficial when populations required for clearance and repair are being lost. By contrast, macrophages that persist in inflammatory states may be better addressed through selective reprogramming or removal rather than indiscriminate cytoprotection. Therapeutic objectives should be tailored to macrophage state and wound phase rather than framed as universal suppression or induction of ferroptosis. Serial assessment may help determine whether treatment should prevent early macrophage dysfunction or restore macrophage function after chronic inflammation has become established.

Translational studies must also show that local interventions reach macrophages at an effective dose without impairing macrophage functions involved in host antimicrobial defense, such as phagocytosis and microbial killing. Release kinetics, retention within the wound, tissue penetration, effects on other cells involved in repair, and compatibility with debridement, infection control, revascularization, and offloading will require evaluation. Improved wound closure alone will not demonstrate a macrophage-mediated mechanism; clinical endpoints should be accompanied by evidence of changes in macrophage iron handling, phospholipid peroxidation, viability, and function. The value of targeting macrophage ferroptosis will depend on whether an intervention preserves beneficial macrophage functions, avoids disruption of host defense, and improves healing beyond standard care.

Taken together, current evidence suggests that macrophage ferroptotic susceptibility and injury may represent one link between the diabetic wound environment, persistent inflammation, and impaired tissue repair. However, direct evidence of ferroptotic death in defined macrophage populations within human DFUs remains limited, and the consequences of macrophage ferroptotic injury are likely to vary according to macrophage state and wound phase. Future studies should combine spatial identification of macrophage populations with biochemical evidence of iron-dependent phospholipid peroxidation and functional rescue. Therapeutic development should therefore prioritize the selective modulation of macrophage ferroptotic vulnerability. Locally delivered ferroptosis inhibitors and bioactive materials with antioxidant or ferroptosis-modulating properties should be evaluated for their ability to achieve this goal while preserving macrophage functions required for antimicrobial defense, inflammatory resolution, and tissue repair.
